# Supplementary material for: Long operating lifetime mid-infrared LEDs based on black phosphorus
Source: Nat Commun. 2023 Aug 10;14:4845. doi: 10.1038/s41467-023-40602-5 (PMC10415361; doi:10.1038/s41467-023-40602-5)
Supplement: Supplementary file 1 — Supplementary Information [file 41467_2023_40602_MOESM1_ESM.pdf]

Supplementary Material for

Long operating lifetime mid-infrared LEDs based on black phosphorus

Naoki Higashitarumizu[1,2,3†], Shogo Tajima[1,3†], Jongchan Kim[1,2,3,††], Mingyang Cai[1,3], and Ali Javey[1,2,3,]*

[1]Electrical Engineering and Computer Sciences, University of California, Berkeley, CA 94720, USA

[2]Materials Sciences Division, Lawrence Berkeley National Laboratory, Berkeley, CA 94720, USA

[3]Berkeley Sensor & Actuator Center, University of California, Berkeley, CA 94720, USA

[†]These authors contributed equally

[††]Present Address: Department of Integrated Display, Engineering, Yonsei University, Seoul, 03722, Republic of Korea

*Address correspondence to: ajavey@berkeley.edu

This PDF file includes:

Supplementary Note 1

Supplementary Figures 1–10

Supplementary Note 1 | Calibrated external QE and internal PL QY measurements.

PL and EL spectra were measured using a customized FTIR spectrometer (iS50, Thermo Fisher) with a liquid N_2-cooled HgCdTe detector.[1,2] A 4.05-μm quantum cascade laser (QF4050T1, Thorlabs) was used as a reference light source to calibrate the PL QY of BP.[1] The ratio of the injected laser power and signal counts in the HgCdTe detector was measured using a spectralon reflection standard (Labsphere) and high-sensitivity thermal power sensor (Ophir) at the focal plane. As the PL/EL emission wavelength ($\lambda \sim 3.6$ μm) is different from that of the calibration laser ($\lambda = 4.05$ μm), the instrument response function (IRF) of the HgCdTe detector was taken into consideration. Also, we considered the difference in angular distribution between the PL/EL emission and the Lambertian emitter. The output power of PL/EL emission (P) was calculated from the detected PL/EL intensity (I) as follows,

$$P = I \times \frac{\text{Laser power}}{\text{Detected intensity}} \times \frac{\text{IRF}\ (\lambda_{\text{PL/EL}})}{\text{IRF}\ (\lambda_{4.05\ \mu\text{m}})} \times \frac{\text{Angular collection}_{\text{PL/EL}}}{\text{Angular collection}_{\text{laser}}} \quad (1)$$

where $\text{IRF}\ (\lambda_{\text{PL/EL}})$ and $\text{IRF}\ (\lambda_{4.05\ \mu\text{m}})$ are the instrument response function of the HgCdTe detector at the PL/EL emission wavelength and at 4.05 μm, respectively; and $\text{Angular collection}_{\text{PL/EL}}/\text{Angular collection}_{\text{laser}}$ are the ratio of collected signal of emission from BP and the Lambertian emitter, with a specific collection angle in the reflective objective. The angular distributions were calculated using FDTD simulations package (FDTD Solutions, Lumerical) for each structures.[1,2] The external quantum efficiency of PL/EL emission was calculated by dividing the number of extracted emitted photons by the number of injected photons/carriers. To calculate internal PL QY, the number of emitted photons by the number of absorbed photons, with using light outcoupling and incoupling efficiencies.[2]

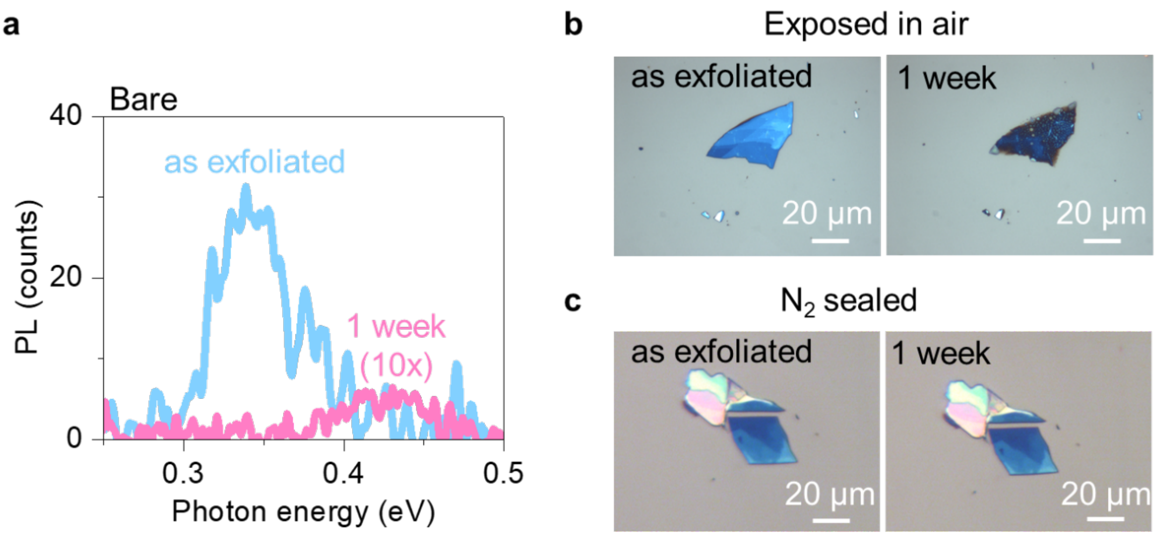

Supplementary Figure 1 | Air stability of BP flakes. a Typical PL spectra from bare sample, as exfoliated and air exposed for 1 week. **b,c** Optical images of BP flakes: bare and N_2 sealed BP, respectively. The BP thickness was fixed at ~10 nm. All the samples were exposed in the air for one week with a relative humidity of 50±5% under dark condition at RT. For the N_2 sealed sample, optical microscopic images were taken inside the glove box to prevent the exposure to the air.

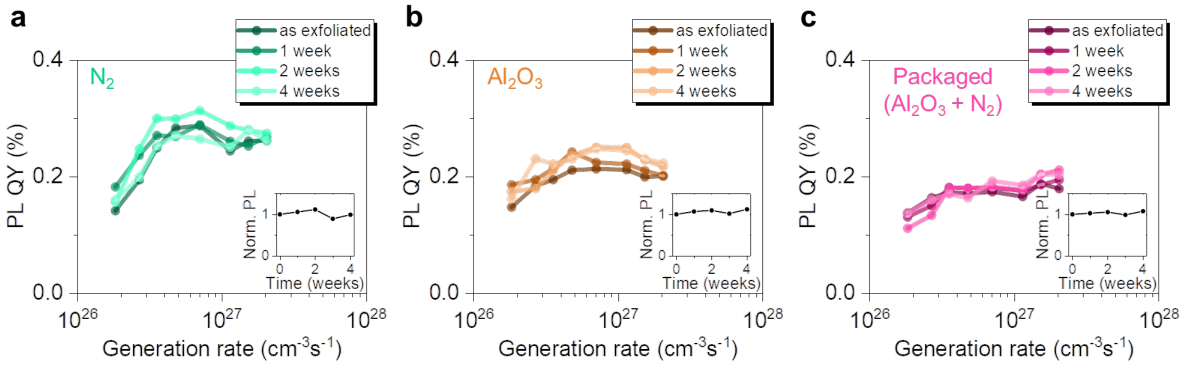

Supplementary Figure 2 | Air stability of PL QY in BP. a–c PL QY in BP as a function of generation rate before and after air exposure, with different conditions: N_2 sealed **a**, with Al_2O_3 passivation **b**, and packaged (Al_2O_3 + N_2) **c**. Inset: normalized average PL intensity as a function of time. The BP thickness was fixed at ~10 nm. All the samples were exposed in the air for one week with a relative humidity of 50±5% under dark condition at RT.

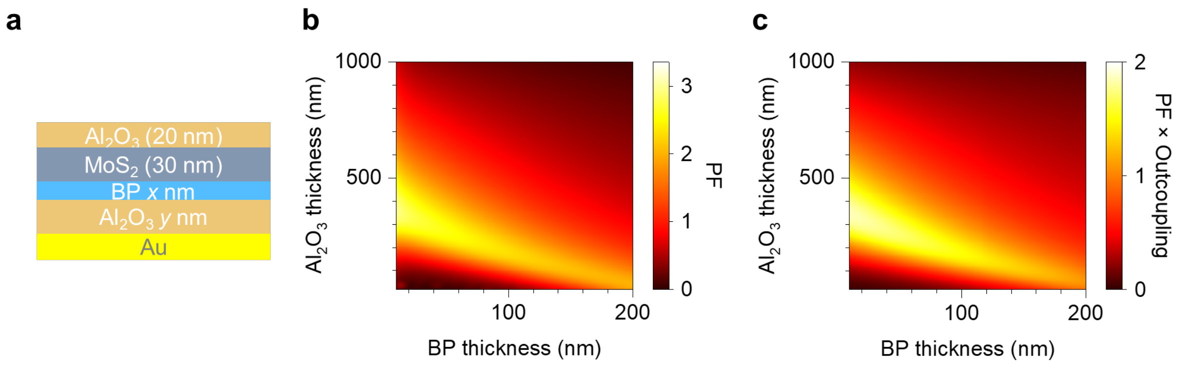

Supplementary Figure 3 | FDTD simulation of optical coupling. a Schematic of simulated structure. **b,c** Simulation results of Purcell factor (PF) and light extraction efficiency, respectively, as a function of BP and top Al_2O_3 thicknesses. The extraction efficiency was calculated as PF × Light outcoupling efficiency.

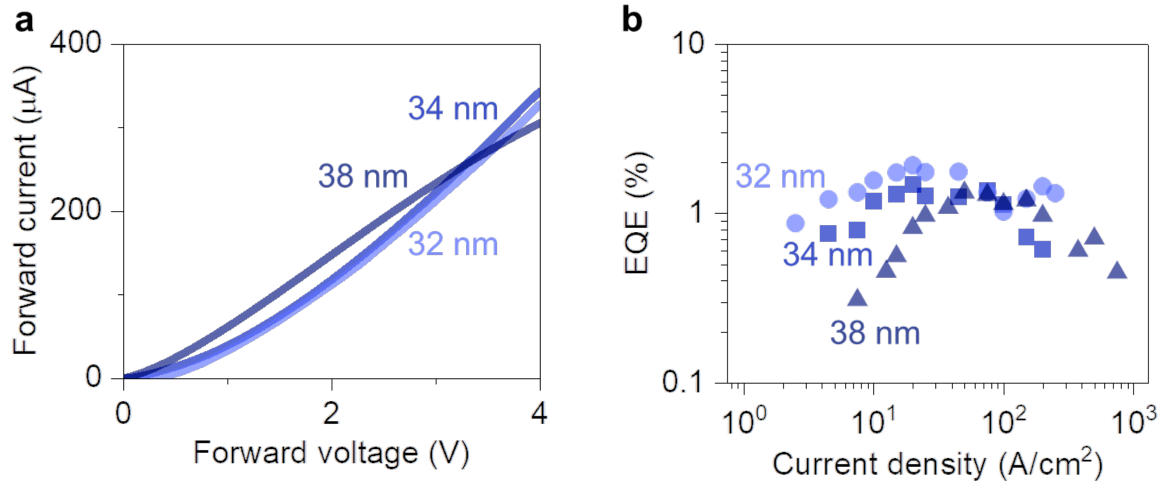

Supplementary Figure 4 | LED performance with different BP thicknesses. a,b *I*–*V* curves and external quantum efficiencies (EQE), respectively, for MoS_2/BP LED with different BP thicknesses of 32 nm (circle), 34 nm (rectangle), and 38 nm (triangle). MoS_2 thickness was fixed at 30 nm.

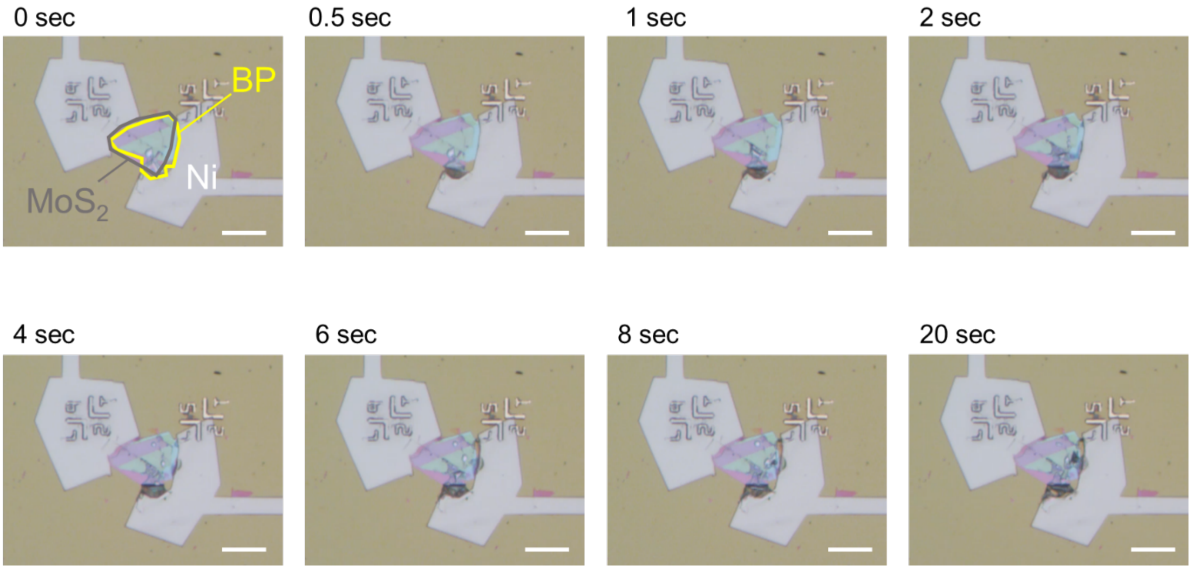

Supplementary Figure 5 | Time-resolved optical microscopic images of LED degradation. BP LED was operated in the air at the current density of ~75 A/cm^2. The scale bar is 40 µm. The degradation starts from the air exposed BP region (see details in Supplemental Movie 1).

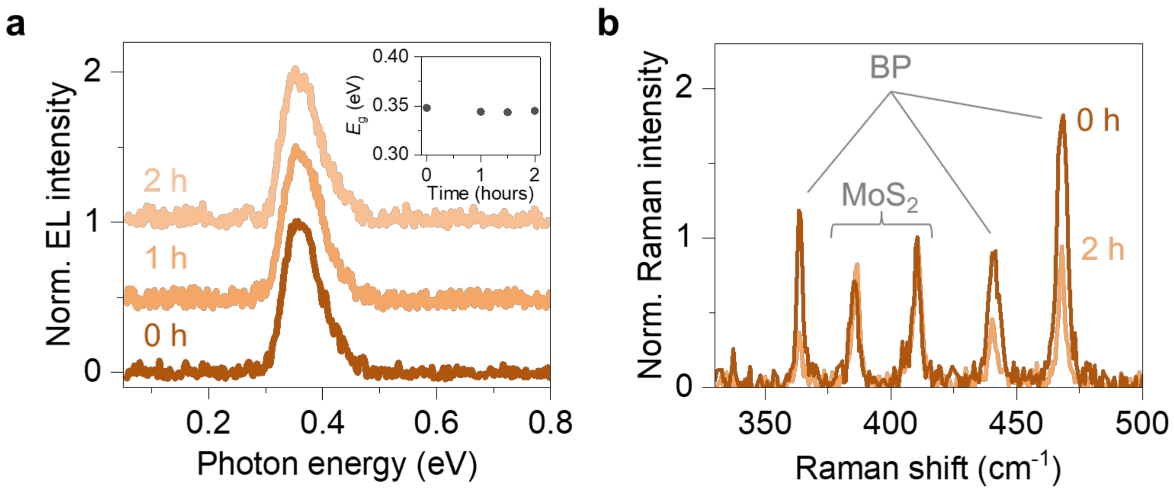

Supplementary Figure 6 | Time evolution of optical characteristics in BP LED during degradation at RT. a Normalized EL spectra of Al_2O_3 passivated BP LED with different operation time of 0–2 hours. Inset: optical bandgap (E_g) as a function of time. No peak shift or additional peak were observed. **b** Normalized micro-Raman spectra of as fabricated and degraded LED devices after two hours operation. External laser was focused on the MoS_2/BP heterostructure. The Raman spectra were normalized with Raman peak from MoS_2 at ~410 cm^{-1}. The Raman signals from BP decreased after two hours compared to MoS_2.

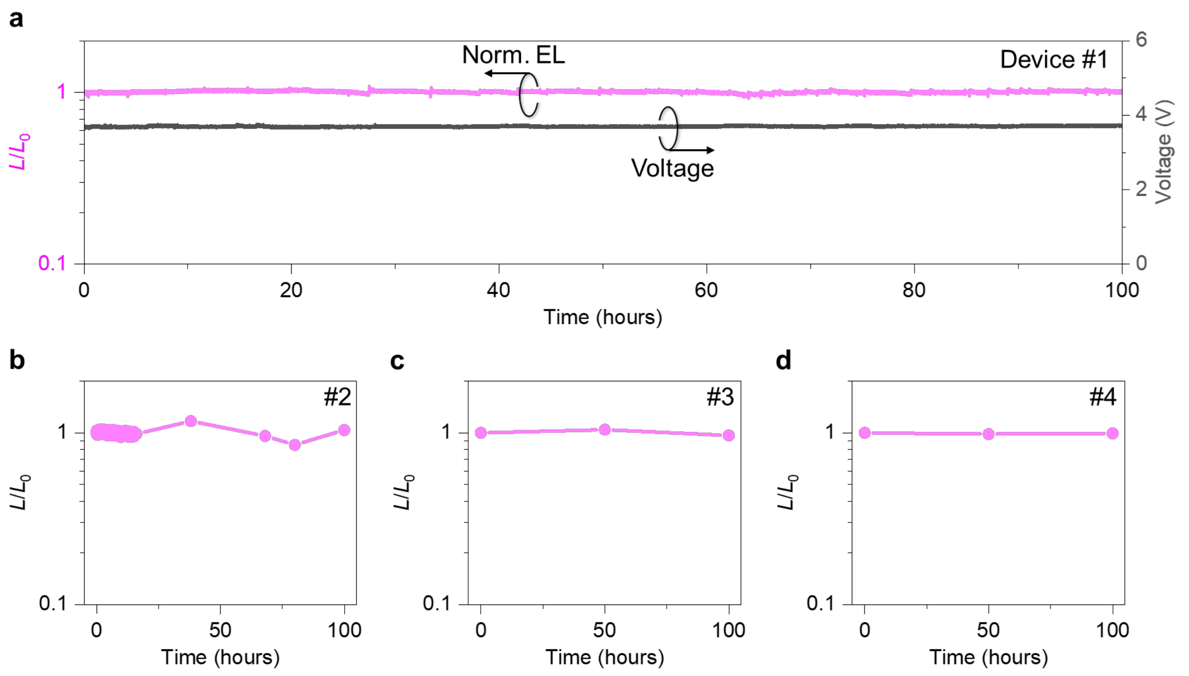

Supplementary Figure 7 | Stable operation of multiple packaged BP LEDs at room temperature. a–d Packaged LEDs were operated in the air at the fixed current density of ~75 A/cm^2. EL intensity and operation voltage remained constant for at least 100 hours, as shown in Supplementary Figure 7a.

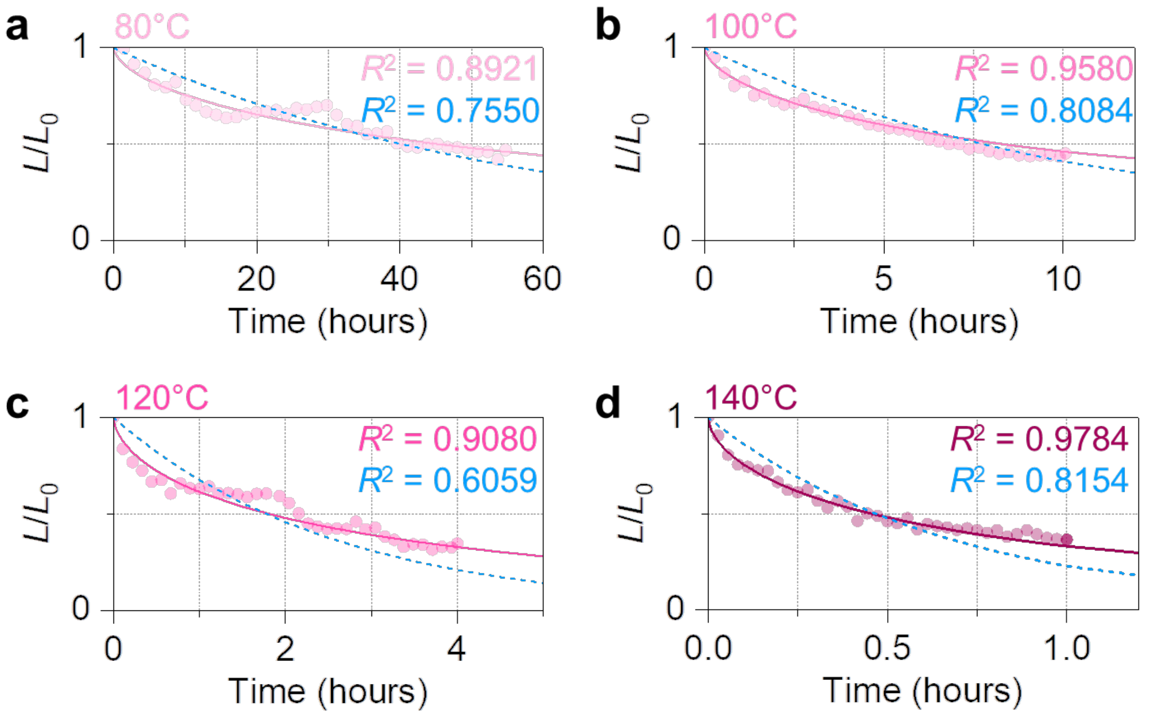

Supplementary Figure 8 | Comparison of fitting functions. a–d Luminance decay of BP LED at different temperature, 80°C, 100°C, 120°C, and 140°C, respectively. Scatters shows experimental results; and solid and dashed lines show fitting curves based of stretched exponential decay model and single exponential model, respectively.

An exponential decay model is the most basic equation to fit the LED aging overtime,[3,4]

$$\frac{L_0}{L} = \exp(-\alpha t) \qquad (2)$$

where t is the LED operation time in the unit of hours; and α is a temperature dependent parameter, which can be written as follows according to the Arrhenius equation,

$$\alpha = A\exp\left(-\frac{E_a}{kT}\right) \qquad (3)$$

where A is the pre-factor, E_a is the activation energy, and k is the Boltzmann constant. By using Equation (1) and (2), experimental results of acceleration lifetime test can be globally fitted with two fitting parameters, A and E_a. However, this single exponential decay model resulted in poor fittings as shown Supplementary Figure 8. A better fitting is provided by a stretched exponential decay model commonly used to fit the LED luminous decay,[3,5]

$$\frac{L_0}{L} = \exp\left[-(\alpha t)^{\beta}\right] \qquad (4)$$

where β is a constant independent of initial EL intensity. Based on Equations (3) and (4), the fitting curves well reproduced the experimental results, as shown in Supplementary Figure 8.

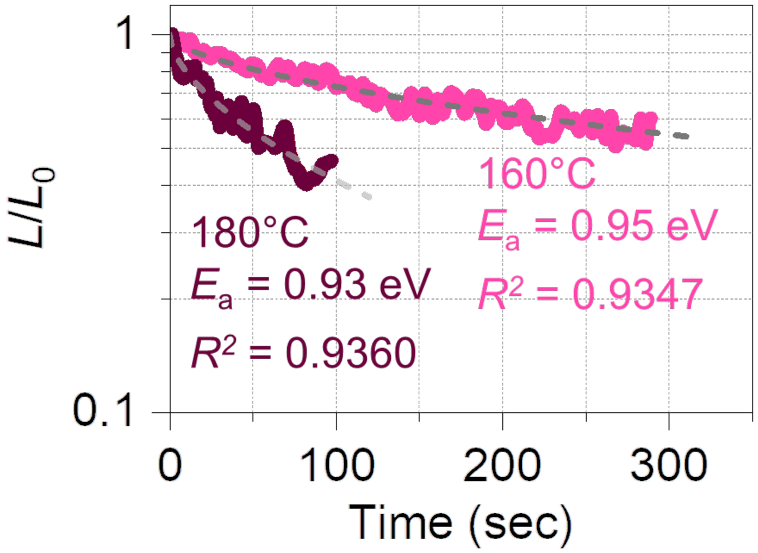

Supplementary Figure 9 | Accelerated lifetime measurement at high temperature regime. Packaged LEDs were operated in the air at 160°C and 180°C. The current density was fixed at ~75 A/cm^2. Dashed lines show fitting curves of the stretched exponential decay model with a fitting parameter of activation energy E_a. The stretching constant β and pre-factor A of Equations (2) and (3) in the main manuscript were fixed at 0.60 and 6.6×10^{11}, respectively, which were obtained from the global fitting at the temperature range of 80–140°C. The activation energies at 160°C and 180°C were obtained to be 0.95 and 0.93 eV, respectively, lower than the activation energy at 80–140°C (E_a = 0.96 eV), which suggest that degradation mechanism above 160°C is different from the lower temperature.

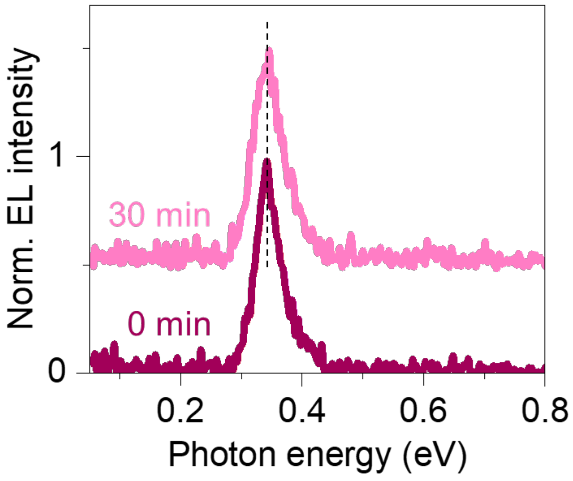

Supplementary Figure 10 | PL spectra of packaged LED at RT after the high-temperature degradation. PL spectra before and after the lifetime test at 140°C for 30 min. The PL spectra were measured at room temperature. No peak shift or additional peak were observed after the degradation, similar to the results for LEDs with Al_2O_3 passivation (Supplementary Figure 6).

Supplementary References

1. Kim, H. *et al.* Actively variable-spectrum optoelectronics with black phosphorus. *Nature* **596**, 232–237 (2021).
2. Higashitarumizu, N. *et al.* Anomalous thickness dependence of photoluminescence quantum yield in black phosphorous. *Nat. Nanotechnol.* **18**, 507–513 (2023).
3. Woo, S.-J., Kim, J. S. & Lee, T.-W. Characterization of stability and challenges to improve lifetime in perovskite LEDs. *Nat. Photonics* **15**, 630–634 (2021).
4. Koh, S. *et al.* Product level accelerated lifetime test for indoor LED luminaires. in *2013 14th International Conference on Thermal, Mechanical and Multi-Physics Simulation and Experiments in Microelectronics and Microsystems (EuroSimE)* 1–6 (IEEE, 2013).
5. Féry, C., Racine, B., Vaufrey, D., Doyeux, H. & Cinà, S. Physical mechanism responsible for the stretched exponential decay behavior of aging organic light-emitting diodes. *Appl. Phys. Lett.* **87**, 213502 (2005).
