# Supplementary material for: Long operating lifetime mid-infrared LEDs based on black phosphorus
Source: Nat Commun. 2023 Aug 10;14:4845. doi: 10.1038/s41467-023-40602-5 (PMC10415361; doi:10.1038/s41467-023-40602-5)
Supplement: Supplementary file 3 — Description of Additional Supplementary Files [file 41467_2023_40602_MOESM3_ESM.pdf]

Description of Additional Supplementary Files

File Name: Supplementary Movie 1

Description: Time evolution of degradation for bare BP LED operated in air
